# Dysregulated apoptosis and NFκB expression in COPD subjects

**DOI:** 10.1186/1465-9921-10-24

**Published:** 2009-03-18

**Authors:** Vanessa Brown, J Stuart Elborn, Judy Bradley, Madeleine Ennis

**Affiliations:** 1 Respiratory Research Group, Centre for Infection and Immunity, School of Medicine, Dentistry and Biomedical Sciences, Queen's University Belfast, Belfast, UK; 2 Institute of Rehabilitation Studies, University of Ulster, Coleraine, UK

## Abstract

**Background:**

The abnormal regulation of neutrophil apoptosis may contribute to the ineffective resolution of inflammation in chronic lung diseases. Multiple signalling pathways are implicated in regulating granulocyte apoptosis, in particular, NFκB (nuclear factor-kappa B) signalling which delays constitutive neutrophil apoptosis. Although some studies have suggested a dysregulation in the apoptosis of airway cells in chronic obstructive pulmonary disease (COPD), no studies to date have directly investigated if NFκB is associated with apoptosis of airway neutrophils from COPD patients. The objectives of this study were to examine spontaneous neutrophil apoptosis in stable COPD subjects (n = 13), healthy smoking controls (n = 9) and non-smoking controls (n = 9) and to investigate whether the neutrophil apoptotic process in inflammatory conditions is associated with NFκB activation.

**Methods:**

Analysis of apoptosis in induced sputum was carried out by 3 methods; light microscopy, Annexin V/Propidium iodide and the terminal transferase-mediated dUTP nick end-labeling (TUNEL) method. Activation of NFκB was assessed using a flow cytometric method and the phosphorylation state of IκBα was carried out using the Bio-Rad Bio-Plex phosphoprotein IκBα assay.

**Results:**

Flow cytometric analysis showed a significant reduction in the percentage of sputum neutrophils undergoing spontaneous apoptosis in healthy smokers and subjects with COPD compared to non-smokers (p < 0.001). Similar findings were demonstrated using the Tunel assay and in the morphological identification of apoptotic neutrophils. A significant increase was observed in the expression of both the p50 (p = 0.006) and p65 (p = 0.006) subunits of NFκB in neutrophils from COPD subjects compared to non-smokers.

**Conclusion:**

These results demonstrate that apoptosis is reduced in the sputum of COPD subjects and in healthy control smokers and may be regulated by an associated activation of NFκB.

## Background

Chronic obstructive pulmonary disease (COPD) is characterised by an inflammatory infiltrate consisting mainly of neutrophils [1], with increased neutrophils and inflammatory mediators in both bronchial tissue and airways of COPD patients [2-4]. In several acute and chronic neutrophilic diseases, such as cystic fibrosis and acute respiratory distress syndrome (ARDS) there is a delay in neutrophil apoptosis resulting in persistent inflammation [5,6]. Studies investigating neutrophil apoptosis in COPD have mainly focussed on circulating neutrophils and shown a reduction in spontaneous apoptosis during an exacerbation of COPD that increases with treatment [7] or no changes in the rate of apoptosis of cultured blood neutrophils between stable COPD subjects, healthy smokers and healthy control non-smokers [8]. The only study to date investigating spontaneous neutrophil apoptosis in sputum from COPD subjects was unable to identify any differences in apoptosis from moderate to severe disease compared to controls [9]. However this study only used one method to directly assess spontaneous neutrophil apoptosis, and since the identification of cells that have clearly adopted an apoptotic phenotype often requires more than one method for determining apoptosis, it has been recommended to use at least two methods to measure apoptosis in order to confirm data [10]. Other studies investigating apoptosis-related factors in COPD have shown that plasma levels of soluble Fas, an inhibitor of apoptosis, are only increased in patients with severe COPD, while plasma levels of the inducer of apoptosis, sFasL, appear unchanged with disease severity [11,12]. Since the transcription factor NFκB controls the expression of many inflammatory and apoptotic genes [13-15], it is of particular interest in a disease such as COPD where neutrophil inflammation persists. The activated form of NFκB is a heterodimer, usually made up of 2 Rel family proteins, p65 (RelA) and p50 (NFκB) subunits. In unstimulated cells NFκB is sequestered in the cytoplasm due to its binding to IκBα and IκBβ. Following cell stimulation, IκB is rapidly phosphorylated by specific protein kinases leading to proteolytic degradation and allowing NFκB to translocate to the cell nucleus where it binds to specific κB recognition elements in the promoter region of target genes (reviewed in [16]). Studies investigating NFκB expression in COPD subjects provide evidence for an increase in NFκB translocation in lung tissue and sputum from COPD subjects compared to non-smoking controls, which appears to be associated with an exacerbation [17-19]. A recent study, described how a number of transcription factors, including NFκB, are overexpressed in bronchial epithelium from smokers with COPD [20].

In order to elucidate the degree of constitutive neutrophil apoptosis in the inflammatory airways of COPD subjects and the potential involvement of NFκB in this process we used three methods to investigate neutrophil apoptosis in COPD and determine any associated increase in NFκB activation in airway neutrophils. We hypothesised that there is a delay in neutrophil apoptosis in COPD subjects that is mediated, in part, by NFκB binding in neutrophils.

## Methods

### Patients

Age-matched stable COPD patients (n = 13), healthy control smokers (n = 9) and control non-smokers (n = 9) were included in the study (all subjects were over 50 years). COPD subjects had a history consistent with COPD as described in the British Thoracic Society guidelines [21]. Inclusion criteria for stable COPD subjects were as follows: no exacerbation within the preceding 4 weeks, over 50 years; ≥ 20 packyear history of smoking; FEV_1_<70% predicted, FEV_1_/FVC ratio < 70% and <15% reversibility in response to β_2 _agonists. They were receiving treatment with anticholinergic, β_2_-agonists and/or steroids. Exclusion criteria were: long-term oral corticosteroids; lung neoplasm and other serious concomitant disease.

Control healthy smokers (HS) were over 50 years and ≥ 20 packyear history of smoking; control non-smokers (NS). Both control groups had normal lung function and subjects were excluded if there was evidence of atopy/asthma, chest disease, recent respiratory tract infection or antibiotic/steroid treatment. The Queen's University Belfast Research Ethics Committee approved the study. Written informed consent was obtained from all participants.

### Sputum induction/processing

Sputum induction was performed using the Sonix 2000 nebuliser (Clement Clarke International Ltd, Harlow, UK) using a method described previously [22,23]. Briefly, 3% saline was nebulised for 20 minutes. Forced expiratory volume in 1 second (FEV_1_) and forced vital capacity (FVC) (Vitalograph) were determined by spirometry according to ATS standards. Nebulisation was stopped if FEV_1 _fell by >20% at any stage. Sputum samples were processed within 2 hours adapting methods described by Pavord *et al*. [22]. The supernatant was removed from the cell pellet and stored at -70°C for future analysis. Cytospin slides were made and stained with Diff Quik (Clin-Tech Ltd, Essex, UK).

### Inflammatory mediators

Sputum CRP concentrations were measured with an ELISA according to the manufacturer's instructions (Bender MedSystems, Austria). Sputum concentrations of IL-6, IL-8 and GM-CSF were determined using a Bioplex multiplex assay kit (Bio-Rad, Hercules, CA) and the Cytokine Reagent kit (Bio-Rad) according to the manufacturer's protocol.

### Measurement of apoptosis

Apoptotic neutrophils were identified by characteristic morphological changes (condensation of nuclear material, 'rounding' of nucleus, shrinkage and pallor of cytoplasm and shrinkage of the cell) [24,25] and expressed as a percentage of total neutrophils counted.

Apoptosis was analysed flow cytometrically using 2 techniques: (1) Samples were labelled with Annexin V (Av) and propidium iodide (PI), following manufacturer's staining protocol, to quantify the percentage of cells undergoing apoptosis and/or necrosis (Pharmingen, San Diego, USA). Analysis was performed using a Coulter Epics Elite and Epics XL flow cytometer (Beckman Coulter, UK Ltd). Samples were gated on the granulocyte population using a forward scatter and side scatter plot with a minimum of 5,000 gated events samples gated. Controls included to set-up compensation and quadrants were: cells stained with Annexin-V FITC alone and cells stained with PI alone. To eliminate cellular debris from the analysis the discrimination level was set at 100. Cells were defined as apoptotic (AV^+^/PI^-^), secondary necrotic (AV^+^/PI^+^), necrotic (AV^+^/PI^-^), or neither apoptotic nor necrotic (AV^-^/PI^-^). Each subpopulation was expressed as a percentage of the total population of granulocytes, (2) The *In situ *cell death detection kit (Roche Diagnostics GmbH, Germany) was used according to the manufacturer's instructions to determine the extent of DNA fragmentation and the proportion of apoptosis. Analysis was performed using a Coulter Epics Elite and Epics XL flow cytometer (Beckman Coulter, UK Ltd). Samples were gated on the granulocyte population as described above. Negative control consisted of cells without incubation with terminal transferase, and DNase I was used as a positive control. Apoptotic cells (i.e. those cells with DNA strand breaks) were identified as fluorescein-dUTP positive (FL1: fluorescent-1).

Plasma concentrations of sFas-L, an inducer of apoptosis, and sFas, an inhibitor of apoptosis, were measured using commercially available ELISA assays (IDS Ltd. UK).

### Quantification of NFκB activation and IκBα phosphorylation

The percentage of granuloctyes positive for the p50/p65 subunits of NFκB in induced sputum was determined using an adapted published flow cytometric method [26]. Nuclei were isolated using the CycleTest PLUS DNA Reagent Kit (BD Biosciences) according to the manufacturer's instruction and stained with anti-human NFκB (p50 or p65) antibody (Santa Cruz Biological, Germany) or isotype-matched control (Southern Biotech, USA), followed by anti-rabbit FITC conjugate (Sigma-Aldrich, Inc. USA) and then propidium iodide (PI) solution. Localisation of granulocyte sputum nuclei in the FS/SC dot plot was determined using previously separated granulocyte nuclei. Acquisition of stained nuclei (minimum 5000 events) was carried out on an Epics Elite and Epics XL flow cytometer. Single nuclei were gated on the basis of PI staining (FL-3 measured at 630 nm), after doublet elimination by FL-3 peak versus integral. Positive p50 or p65 expression (determined in the FL-1 plot) was expressed as percentage of granulocyte nuclei.

Protein lysates were prepared by using Cell lysis kit (Bio-Rad, USA) and the presence of phosphorylated IκBα was detected by Bio-Plex Phospho-IκBα (Ser32/Ser36) assay kit (Bio-Rad, USA) according to the manufacturer's protocol. Data were collected and analysed using the Bio-Plex suspension array system (Bio-Rad Laboratories USA).

### Statistical analysis

Statistical analysis was performed using SPSS version 13.0 (SPSS inc., Chicago, IL, USA). Comparisons between groups were performed with the one-way ANOVA followed by Tukey's post-hoc test or the nonparametric Kruskal-Wallis test followed by Dunn's post-hoc test when the data were not normally distributed. Direct comparisons between two groups were performed with the nonparametric Mann-Whitney test. A p value of less than 0.05 was regarded as significant.

## Results

Demographics are summarised in Table 1. Sputum induction was not successful in all subjects; a total of 14 COPD, 13 healthy smokers and 12 non-smoking controls were initially recruited for the study, with 13, 9 and 9 subjects respectively producing an adequate sample to proceed with experiments. Furthermore some subjects did not produce enough for all analyses to be carried out and hence numbers between groups may vary.

**Table 1:** Demographic and lung function data

	**COPD** **n = 13**	**Healthy smokers (HS)** **n = 9**	**Non-smokers(NS)** **n = 9**
**Age**	68.38 (1.86)	61.56 (3.13)	68.67(1.90)
**Sex (M/F)**	7/6	6/3	1/8
**Smoker (current/ex)**	5/7	9/0	0/2
**Pack Years**	49.08 (10.01)	42.28 (5.68)	
**FEV**_1_**(L)**	0.88 (0.12)	2.55 (0.16)	1.97 (0.12)
**FEV1%**	36.25 (3.94)	90.22(4.13)	93.75 (9.22)
**FVC (L)**	1.77 (0.1)	3.56 (0.18)	2.43 (0.12)
**FVC %**	59.67 (3.88)	101.2 (5.92)	92.0 (8.27)
**Ratio (FEV/FVC)**	48.98 (5.21)	71.66 (2.90)	81.23 (3.70)

Data are expressed as mean (SEM).

FEV_1_: Forced expiratory volume in the first second.

FVC: Forced vital capacity

Analysis of the differential and total cell counts showed significant differences in the percentage of macrophages, neutrophils and epithelial cells (expressed as a percentage of total cell count) between all 3 groups (Table 2). The percentage of neutrophils was higher in COPD patients compared with healthy smokers and non-smokers (p < 0.05). Conversely the percentage of macrophages was decreased in COPD patients (p < 0.05) compared to healthy smokers and non-smokers.

**Table 2:** Total and differential sputum cell counts

	**COPD**	**Healthy smokers (HS)**	**Non-smokers (NS)**	*p value
**Total cell count (× 10**^6^**/g sputum)**	2.43 (0.7)	3.26 (1.93)	1.32 (0.46)	NS
**% Macrophage**	32.40 (4.30)	47.90 (3.24)	44.28 (3.80)	< 0.05
**% Neutrophil**	58.78 (4.90)	44.66 (3.02)	41.35 (4.32)	< 0.05
**% Eosinophils**	3.27 (1.79)	0.83 (0.16)	1.92 (0.84)	NS
**% Lymphocytes**	3.54 (0.86)	3.17 (0.64)	8.0 (3.31)	NS
**% Epithelial cells**	0.76 (0.30)	1.80 (0.32)	0.72 (0.28)	< 0.05
**% Squamous cells**	1.27 (0.53)	1.66 (0.54)	2.04 (0.81)	NS

Data are expressed as mean (SEM).

* Comparison between the 3 groups using one-way ANOVA.

NS: not significant

### Inflammatory mediators: C-reactive protein (CRP) and cytokines

**Figure 1:**
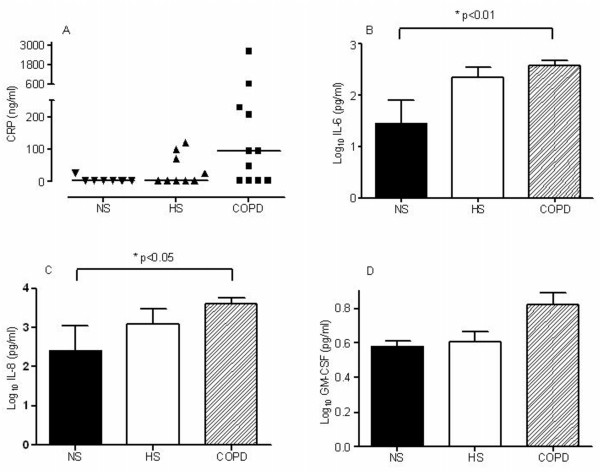
**(a) CRP levels in sputum from non-smokers, n = 7 (NS), healthy smokers, n = 9 (HS) and in COPD subjects, n = 12**. **(b) **Log_10 _IL-6 levels (pg/ml) in sputum from non-smokers, n = 5 (NS), healthy smokers, n = 5 (HS) and in COPD subjects, n = 12 **(c) **IL-8 levels (pg/ml) in sputum from non-smokers, n = 5 (NS), healthy smokers, n = 5 (HS) and in COPD subjects, n = 12 **(d) **Log_10 _GM-CSF levels (pg/ml) in sputum from non-smokers, n = 5 (NS), healthy smokers, n = 5 (HS) and in COPD subjects, n = 12. Data are expressed as mean (SEM). p values shown is from Tukey's multiple comparison post-hoc analysis following one-way ANOVA.

To investigate airway inflammation the levels of the acute-phase reactant C-reactive protein, CRP, IL-6, IL-8 and GM-CSF were measured (Figure 1). In sputum there was a median 30-fold increase in CRP in COPD subjects compared to both healthy smokers and non-smokers and concentrations of IL-6 and IL-8 in sputum were significantly higher in COPD subjects compared to non-smokers. GM-CSF levels were higher, but not statistically different, in COPD subjects compared to healthy smokers and non-smokers.

### Quantification of neutrophil apoptosis

**Figure 2:**
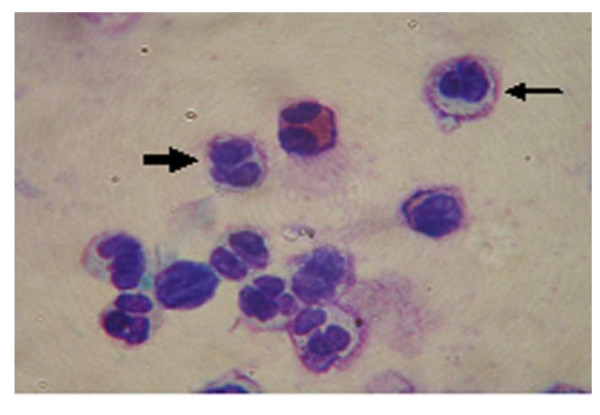
**Apoptotic sputum neutrophils identified using light microscopy**. Typical apoptotic neutrophils displaying loss of chromatin filaments (heavy arrow) and shrinkage of the nucleus (thin arrow).

The results from the three different methods used to assess apoptosis are summarised in Table 3. Using one-way ANOVA test, the percentage of apoptotic cells were significantly different between all 3 assays (p < 0.0001) with post-hoc test showing a higher percentage of Tunel positive cells, Av+/PI+ and Av-/PI+ cells compared to the percentage of neutrophils showing morphological features of apoptosis (Figure 2) (p < 0.001, p < 0.001 and p < 0.05 respectively). Also there was a higher percentage of apoptotic neutrophils detected by the Tunel assay compared with those cells that were Av+/PI- (p < 0.0.5). There was a significant correlation between Av+/PI- neutrophils (early apoptotic neutrophils) and the percentage of neutrophils displaying morphological features of apoptosis (r = 0.67, p < 0.001, Pearson correlation).

**Table 3:** Comparison of apoptotic assays from sputum neutrophils

	**Method 1**	**Method 2**			**Method 3**
	**% Apoptotic neutrophils**^1^	**% Av**^+^**/PI**^-^	**% Av**^+^**/PI**^+^	**% Av**^-^**/PI**^+^	**% Tunel** **+ve cells**^2^

**ALL SUBJECTS**	10.9 (1.22)	17.19 (2.85)	37.63 (5.27)	21.67 (4.02)	28.56 (4.09)

Method 1: Microscopic morphological identification. Method 2: Annexin V/Propidium iodide (PI) staining. Method 3: Identification of DNA fragmentation (Tunel assay).

% AV^+^/PI^-^: early apoptosis, % AV^+/^PI^+^: later stage apoptosis/secondary necrosis and % AV^-^/PI^+^: necrosis.

^1^Significantly different compared to Tunel +ve cells (p < 0.001), % AV^+^/PI^+ ^(p < 0.001) and % AV^-^/PI^+ ^neutrophils using Tukey's multiple comparison post-hoc test. ^2^Significantly different compared to % AV^+^/PI^- ^cells (p < 0.05).

Data are expressed as mean (SEM)

**Figure 3:**
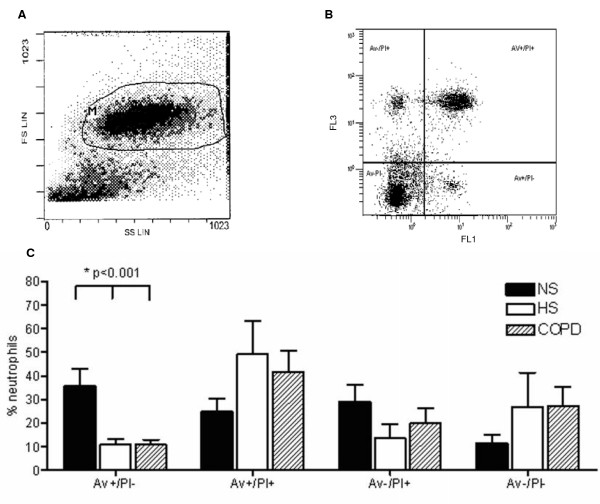
**Annexin V/PI analysis in induced sputum.** (a) Horizontal axis represents side scatter (SS) and vertical axis represents forward scatter (FS) (linear scale). Gated area, containing granulocytes, represented by area marked M **(b) **Horizontal axis represents intensity of staining for Av (Annexin V) (logarithmic scale) and vertical axis intensity of staining for PI (logarithmic scale). Typical dot plot representing populations of early apoptotic (AV^+^/PI^-^), secondary necrotic (AV^+^/PI^+^), and necrotic granulocytes (AV^+^/PI^-^) **(c) **Analysis of neutrophil apoptosis in sputum using the Annexin V/Propidium iodide assay. One way ANOVA showed a reduction in Av^+^/PI^- ^in healthy smokers (HS) and COPD subjects compared to non-smokers (NS), p < 0.001. % AV^+^/PI^-^: early apoptosis, % AV^+/^PI^+^: later stage apoptosis/secondary necrosis:% AV^-^/PI^+^: necrosis.

**Figure 4:**
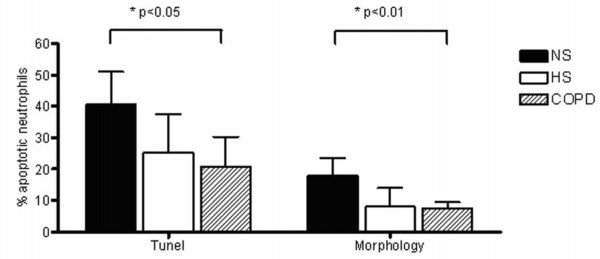
**Percent of apoptotic neutrophils in sputum by DNA strand breaks (Tunel) and morphological features of apoptosis**. Data are displayed as median (interquartile range). Kruskal-Wallis test was used to determine any significant differences between the 3 groups, followed by post-hoc analysis (Dunn's Multiple Comparison test). p values shown are from post-hoc analysis.

Using the Annexin V/PI method for the assessment of apoptosis, granulocytes were gated using the forward and side scatter dot plot (Figure 3a) and assessed for staining of Annexin V and/or PI (Figure 3b). Subjects with COPD and healthy smokers had a statistically significant reduction in early stage apoptosis (as measured by Av+/PI- staining) compared to non-smokers, p < 0.001 (Figure 3c). There was a trend for an increase in late apoptosis/secondary necrosis (Av+/PI+) in subjects with COPD and healthy smokers compared to non-smokers (Figure 3c). Despite differences in methodologies, similar findings to those with the Annexin V/PI assay were seen using both the Tunel assay and the morphological identification of apoptosis (Figure 4). A significant reduction in the percentage of Tunel positive cells was observed in COPD subjects compared to non-smokers and also in the percentage of cells that were morphologically identified as apoptotic using light microscopy (Figure 4).

**Figure 5:**
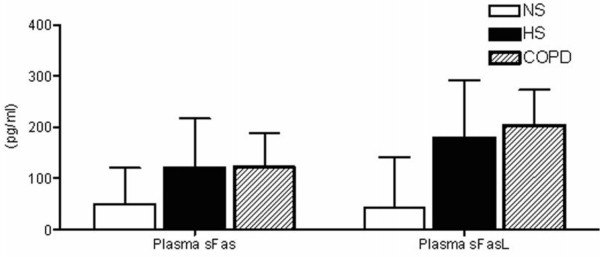
**sFas and sFasL levels in plasma from non-smokers (NS), healthy smokers (HS) and COPD subjects**. Data are displayed as median (interquartile range).

This study also examined the plasma levels of soluble Fas/APO-I receptor (sFas), an inhibitor of apoptosis, and soluble Fas ligand (sFas-L), an inducer of apoptosis, in all three subject groups (Figure 5). Although plasma sFas levels were slightly higher in COPD subjects compared to non-smokers there was no statistical significance between the groups. Similarly assessment of sFasL showed no significant differences between the subject groups.

### IκBα phosphorylation and NFκB activation in induced sputum

**Figure 6:**
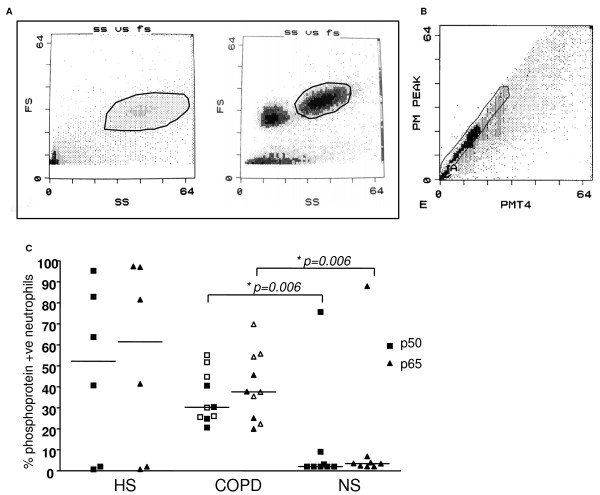
**NF[kappa]B analysis in induced sputum.** (a) Identification of granulocyte nuclei (gated area shown) in sputum by comparing separated granulocyte nuclei from sputum sample (left) with whole sputum nuclei (right) (b) Sputum granulocyte nuclei: FL-3-peak versus FL-3 integral dot plot showing a singlet gate to exclude aggregates (c) Percentage of neutrophils expressing p50 and p65 (NFκB activation) in induced sputum in non-smokers (NS), healthy smokers (HS) and in COPD subjects. In COPD subjects closed symbols = current smokers and open symbols = ex-smokers. Mann-Whitney U test was used to determine any significant differences. COPD n = 12; HS n = 6; NS n = 8. Line represents median value.

Localisation of single granulocyte nuclei allowed for the quantification of NFκB activation in sputum from COPD subjects, healthy smoking controls and non-smoking controls (Figure 6a and 6b). p50 and p65 subunit expression in sputum neutrophil nuclei showed a significant increase in the expression of both NFκB subunits in COPD subjects (p50, p = 0.006 and p65: p = 0.006) compared to non-smokers (Figure 6c). Although there was a higher expression of p50 and p65-positive neutrophil nuclei in healthy smokers compared to non-smokers, this did not reach significance. An association was observed between sFasL in plasma from non-smokers and activation of p65 in sputum (r = -0.90, p = 0.002, Spearman's correlation).

**Figure 7:**
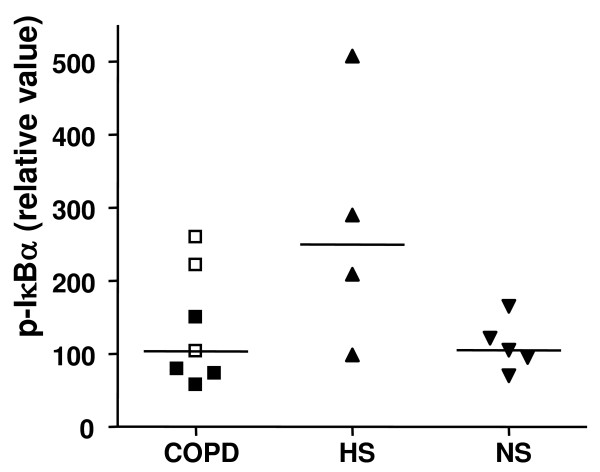
**Quantification of IκBα activation in induced sputum**. Control non-smokers (NS, n = 5), healthy smokers (HS, n = 4) and in COPD subjects (n = 7). In COPD subjects closed symbols = current smokers and open symbols = ex-smokers. Line represents median value.

To further assess the role of NFκB in controlling sputum neutrophil apoptosis in COPD subjects, the phosphorylation of IκBα in sputum was investigated. Although there was a trend towards a higher level of phosphorylated IκBα in healthy smokers compared to non-smokers, this did not reach significance (Figure 7).

## Discussion

In this study, flow cytometric analysis demonstrated that apoptosis is abnormally regulated in COPD subjects that this appears to be, at least in part, regulated by the activation of NFκB. It is postulated that oxidative stress and survival factors contribute to the longevity of the neutrophil that perhaps overwhelms the macrophage clearance mechanism leading to persistent airway inflammation in COPD subjects.

Assays investigating apoptosis should be interpreted with care. Firstly, cells can display morphological features of apoptosis without DNA fragmentation [27,28] and it is also possible that the Tunel assay can generate false-positive results since random DNA fragmentation during necrosis may also generate 3'OH DNA ends [29]. Despite the concept that the morphological detection and quantification of apoptotic cells is regarded as the 'gold standard' method [30], results from this study support the notion that in some instances morphological identification of apoptotic cells may underestimate apoptosis [31]. Therefore it is imperative that at least 2 different methods are used in the quantification of apoptosis, to confirm data and to ensure different stages of apoptosis are investigated. Despite differences in methods, all three assays showed significant reduction in the percentage of apoptotic neutrophils between COPD subjects and non-smokers. Quantification of apoptosis by Annexin V/PI staining also showed a decrease in healthy smoking subjects compared to non-smokers. This study, along with others has reported an increase in airway neutrophils in COPD subjects [2-4,32] and it is plausible that this accumulation of neutrophils may be due to both increased recruitment in the airways and inhibition of apoptosis [33,34].

Our findings show that the percentage of neutrophils in early stage apoptosis are significantly reduced in COPD subjects and in healthy smokers and concomitantly have a higher percentage of neutrophils undergoing secondary necrosis compared to non-smokers. Similar results were shown using by both the Tunel method and by light microscopic morphological identification. Several factors can influence the process of neutrophil apoptosis. While data suggests that corticosteroids inhibit neutrophil apoptosis [35], others have shown that corticosteroids do not affect spontaneous neutrophil apoptosis in COPD patients [7]. More recently work suggests that β 2 agonists alone have negligible effects on neutrophils but together with corticosteroids actually enhances inhibition of apoptosis [36]. One limitation of this study is the lack of investigation of the influence of medication on neutrophil apoptosis. Although COPD treatment included β_2 _agonists, corticosteroids and/or anti-cholinergics, all healthy smokers recruited in this study had not received any recent oral/inhaled steroids and therefore it is likely that the similar inflammatory status observed in COPD and healthy smokers is not a consequence of corticosteroid treatment. Furthermore it has been suggested that *in vitro *findings showing delayed neutrophil apoptosis due to corticosteroids is more than likely overwhelmed by the *in vivo *inhibition of the anti-apoptotic effects of inflammatory cytokines [7]. IL-6, IL-8 and GM-CSF are important cytokines in the regulation of airway inflammation and several studies have shown these cytokines play a key role in the pathophysiology of COPD [37]. *In vitro *studies have shown that the pro-inflammatory cytokines GM-CSF, IL-8 and IL-6 inhibit apoptosis of granulocytes [38-41] and therefore the observed increase in IL-6 and IL-8 in COPD subjects compared to non-smokers may account for the reduced neutrophil apoptosis in COPD subjects.

One potential disadvantage of using induced sputum is the low number of cells and indeed for some patients production of an adequate volume of sputum is virtually impossible. To this end we were limited to measuring Fas and FasL expression in the plasma of all subjects. Fas has been shown to be an important mediator of apoptotic cell death, as well as being involved in inflammation. Results in this study suggest a trend for an increase in sFas, an inhibitor of apoptosis, in plasma from COPD subjects. The strong inverse relationship between sFasL (inducer of apoptosis) in plasma from non-smokers and activation of p65 in sputum observed in this study lends support to the concept that an increase in nuclear IκBα is associated with inhibition of NFκB activation and the subsequent induction of apoptosis [42]. In bronchial epithelium, there is an increase in p65 expression (regarded as NFκB activation) in COPD patients and smokers with normal lung function, compared to normal control subjects [43] and investigations of NFκB activity in sputum from COPD show an increase in NFκB translocation, possibly in macrophages, during an exacerbation in COPD subjects [17,18]. However, the study by Drost *et al. *analysed sputum leukocytes as a whole and therefore does not indicate which cells may be associated with NFκB translocation. It seems that there are differences in the NFκB regulation of apoptosis between neutrophils and other cells, with the inhibition of NFκB inducing apoptosis in murine B cells [44], and in other cell types, activation of NFκB seems to correlate with the onset of apoptosis [45]. The significant increase in percentage of neutrophil NFκB phosphorylation in COPD subjects compared to non-smokers in this study provides evidence to support a role for NFκB translocation and activation in delaying apoptosis in COPD subjects and to date is the only reported study to investigate the activation of this transcription factor and its' inhibitor, IκBα, in neutrophils from induced sputum. A higher expression of p50 and p65-positive neutrophil nuclei was observed in healthy smokers compared to non-smoking controls, however, this did not reach significance, possibly due to the low number of non-smoking controls available and the inter-subject variability. To fully understand the involvement of NFκB in neutrophil apoptosis in COPD, it is important to assess IκB phosphorylation state as this precedes ubiquitination and degradation and subsequent NFκB activation. However, the quantity of cellular lysates varied greatly from patient to patient and restricted analysis of the phosphorylation state of IκBα in all subjects. Therefore a lack of power meant there was no observed statistical difference in IκBα phosphorylation between the three groups.

The reported increase in NFκB induction and activation may be due to the presence of high levels of cytokines which prolong the lifespan of the neutrophil (eg. GM-CSF), the ability of IL-8 and IL-6 (both of which were significantly increased in COPD subjects) to suppress spontaneous apoptosis [46,47] or possibly due to viral and/or bacterial infection. An ongoing bacterial infection may exacerbate airway inflammation in COPD subjects by signalling via Toll-like receptor (TLR) activation to activate NFκB and induce expression of NFκB-regulated genes thereby inhibiting neutrophil apoptosis. It is well known that cigarette smoke is the most important risk factor for COPD, however the exact reasons as to why only a minority of smokers (15–20%) develop clinical COPD remain unknown. This study, although suggesting that the inflammatory status and activation state of neutrophils between COPD subjects and healthy smokers are similar, does not address the issue of increased susceptibility to smoking in COPD development. Previous cross-sectional and longitudinal studies of healthy smokers suggest that although inflammation is partially reversible with smoking cessation or even reduction this is not observed in COPD ex-smokers [48]. Furthermore COPD ex-smokers have more extensive inflammation than asymptomatic ex-smokers, including increased sputum neutrophils [49]. Results from this study suggest that cigarette smoke alone does not promote an "abnormal" inflammatory response in COPD subjects and may suggest that the development of COPD in "susceptible" individuals is due to an interaction between environmental and genetic factors.

Much work investigating the mechanism of cigarette smoke effect on neutrophil infiltration and apoptosis has suggested that exposure to cigarette smoke initiates NFκB activation, potentially in macrophages, that leads to infiltration of neutrophils to the airways *in vivo *[50,51] and the use of acrolein, a toxic aldehyde found in cigarette smoke, in *in vitro *experiments can inhibit an number of important kinases and spontaneous neutrophil apoptosis [52]. Several similarities in the inflammatory response (including neutrophil apoptosis and activation of NFκB) between COPD and healthy smokers in this study may suggest that the observed inflammation is a consequence of smoking. However, the majority of COPD subjects were ex-smokers and therefore support the notion that the inflammatory response in ex-smokers with COPD is due the disease process itself [53] and perhaps it is the remodelling in COPD that maintains this inflammatory process [49].

## Conclusion

In summary, this study provides evidence for a reduction in the percentage of neutrophils that have undergone spontaneous apoptosis in the airways of COPD subjects using morphological identification of apoptosis, quantification of phosphatidylserine exposure and DNA fragmentation. It is likely that the prolonged neutrophil survival reported in this study in COPD subjects may be due to altered expression of genes associated with apoptosis that are controlled by NFκB. The similarity in neutrophil apoptosis and in some inflammatory markers between smokers and subjects with COPD needs to be further investigated with more diverse and larger subject groups to determine factors associated with disease progression versus inflammation associated with smoking *per se*.

## Competing interests

The authors declare that they have no competing interests. 

## Authors' contributions

VB contributed to the design of the study, acquisition of data, analysis and interpretation, and manuscript draft. ME and JSE were responsible for design and management of the study, statistical analysis and manuscript draft. JB participated in the study design and coordination and helped to draft the manuscript. All authors read and approved the final manuscript.
